# Hearing sensitivity in perinatally HIV-infected children

**DOI:** 10.1186/1471-2334-14-S2-P37

**Published:** 2014-05-23

**Authors:** Peter Torre, Alyssa Cook, Hannah Martin, Haley Elliott, Gouwa Dawood, Barbara Laughton

**Affiliations:** 1 San Diego State University, San Diego, USA

## Introduction

Research on the effects of perinatal human immunodeficiency virus (HIV) exposure on hearing sensitivity is increasing. This is important because hearing loss can delay language and communication development in young children. The purpose of this project was to evaluate hearing sensitivity characteristics in HIV+ and HIV- children.

## Materials and methods

Children were recruited from outpatient clinics within Tygerberg Children’s Hospital in Cape Town, South Africa. Caregiver report of middle ear infections was obtained through a questionnaire. Bilateral otoscopy, tympanometry, then pure-tone testing was completed. Hearing loss was defined as a pure-tone average (PTA) of 500, 1000, 2000, and 4000 Hz of >15 decibels of hearing level (dB HL) in the poorer ear. Unilateral hearing loss was defined as one normal ear and one ear with hearing loss. Conductive hearing loss was defined as an air-bone gap of ≥15 dB at a minimum of two frequencies in either ear. The percentage of children with hearing loss and mean PTA were compared between HIV+ and HIV- children. The effect of WHO status on hearing loss was examined for HIV+ children.

## Results

Sixty-one children: 37 HIV+, 24 unexposed uninfected (HIV-), mean age 7.1 years (SD=1.6) were assessed. HIV+ children had a higher rate of reported middle ear infections and more conductive hearing loss. The risk for hearing loss was higher for HIV+ children (p=0.18), who also had a significantly higher mean PTA in the poorer ear than HIV- children (15.1 dB vs 8.6 dB, p=0.03). Six HIV+ children had unilateral hearing loss whereas one HIV- child had a unilateral hearing loss. Risk for hearing loss was higher for HIV+ children with WHO stage IV disease compared to other stages, but not significant.

## Conclusions

There were more middle ear infections, a higher risk of hearing loss, and more unilateral hearing loss in HIV+ than HIV- children; HIV+ children had a significantly higher PTA (worse hearing) in their poorer ear. The risk of hearing loss was higher in those with WHO stage IV disease than those with stage II or III.

